# A large-scale fabrication of flower-like submicrometer-sized tungsten whiskers via metal catalysis

**DOI:** 10.1186/1556-276X-7-325

**Published:** 2012-06-21

**Authors:** Yunzhu Ma, Jing Li, Wensheng Liu, Yubin Shi

**Affiliations:** 1 State Key Laboratory for Powder Metallurgy, Central South University, Changsha, Hunan Province, 410083, People’s Republic of China

**Keywords:** Nanomaterials, Tungsten whisker-like, Metal catalysis-assisted, Flower-like, Growth mechanism, Large scale fabrication

## Abstract

Tungsten powder mixed with an appropriate amount of nickel and iron powders is used as raw material to fabricate large-scale tungsten whisker-like structure. The morphology, microstructure and composition of the whisker-like tungsten are observed and tested by scanning electron microscope and FESEM, transmission electron microscopy, X-ray spectroscopy, and X-ray diffraction, respectively. The main component of the tungsten whisker-like structure is tungsten, which has the axial growth along the <100 > direction with large aspect ratio and possesses flower-like structure. Large-scale submicrometer-sized whisker-like tungsten was fabricated via vapor phase deposition approach with the aid of metal catalysts at 800°C by holding for 6 h in the appropriate atmosphere. The growth procedure of flower-like tungsten whisker is probably based on the vapor–liquid–solid mechanism at beginning of the formation of tungsten nuclei, then vapor-solid mechanism is dominant.

## Background

One-dimensional nanomaterials of tungsten have excellent performance of chemical, physical, electrical, and mechanical properties [1], so metallic tungsten one-dimensional nanomaterials have attracted considerable attention as promising materials for field emitters [2-4], displays [5], sensors [6,7], etc. Many approaches were used to prepare tungsten one-dimensional nanomaterials, such as metal catalysis induction method [8,9], vapor deposition [10,11], template [12] or substrate [13], etching [14,15], sputtering [2] or electron beam-induced deposition [3,16], etc. However, organizing these one-dimensional nanomaterials into highly ordered arrays can be extremely challenging. The key problem in the preparation of tungsten one-dimensional nanomaterials is to control their morphology, size and uniformity effectively at the same time. So, many growth mechanisms had been proposed according to the experiment results to control the growth of tungsten one-dimensional nanomaterials accurately.

It is crucial to understand the tungsten one-dimensional nanomaterial (e.g., nanowires, nanofibers, nanorods, nanoneedles) growth mechanism for the growth control of tungsten nanomaterials accurately. Because of its high melting point, low atomic diffusion rate and other special properties, there are both similarities and differences between the growth mechanism of tungsten nanomaterials and general inorganic nanomaterials. For this reason, the researchers had proposed a number of growth models which could be used for reference, for example, VS model [17,18], vapor–liquid–solid (VLS) model [19-22], VSS model [8,23], etc. The growth mechanism is different with distinct preparation conditions.

In this research, a novel route with low cost was used, and large-scale flower-like submicrometer-sized tungsten whiskers were prepared by the vapor phase deposition method with the aid of metal catalysts. According to the experiment results, the growth model of tungsten whisker-like structure was proposed.

## Methods

The raw materials are tungsten powders (purity, 99.5 wt.%; particle size, 1 to 3 μm), carbonyl iron powders (purity, 99.5 wt.%; particle size, 3 to 5 μm) and carbonyl nickel powders (purity, 99.5 wt.%; particle size, 3 to 5 μm). The mixed powders (weight ratio of W:Ni:Fe equals 93:4.9:2.1) were obtained by wet milling for 2 h, then the mixed powders with a weight of 2.0 g were reacted by holding for 6 h in a horizontal tube furnace at 800°C. The atmosphere was a mixed gas of N_2_, H_2_ and a little water vapor. The flow rates of N_2_ and H_2_ were 0.3 and 0.03 L/min, respectively. The water vapor was brought into the system by the water bath of H_2_, and the water bath temperature was 80°C. The as-products were characterized by X-ray diffraction (XRD), scanning electron microscopy and FESEM, energy dispersive spectroscopy (EDS) and transmission electron microscopy (TEM), respectively.

## Results and discussion

### Characteristics of the submicrometer-sized tungsten whiskers

**Figure 1:**
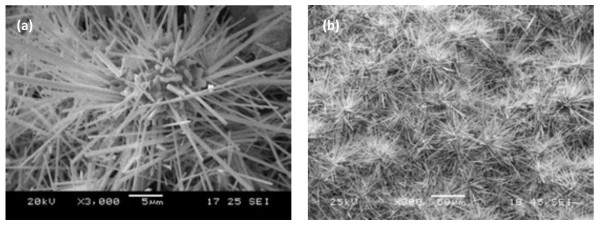
**Morphologies of flower-like tungsten whisker produced.** (**a**) Single-bundle flower-like tungsten whiskers; (**b**) large-scale flower-like tungsten whiskers.

**Figure 2:**
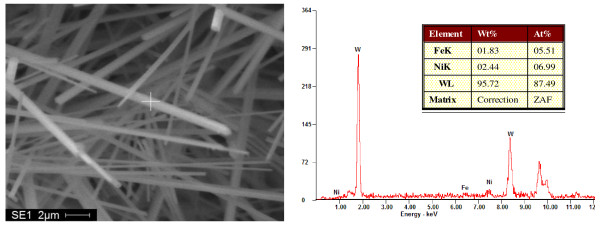
**EDS pattern of whisker-like tungsten produced.** ZAF, Z is atomic number correction factor, A is absorption correction factor, and F is fluorescence correction factor.

**Figure 3:**
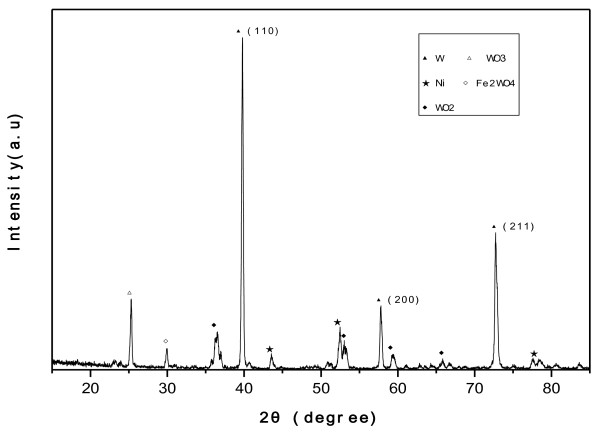
XRD pattern of whisker-like tungsten produced.

Figure1 shows the morphology of as-produced tungsten whiskers. It has a flower-like figuration with a sinter as its growing center. The results indicated that the tungsten whisker-like structure was grown from the sintered powder, and the corresponding EDS pattern was shown in Figure2. The XRD pattern of the as-products (Figure3) indicated that the main phase was tungsten with little tungsten oxide.

**Figure 4:**
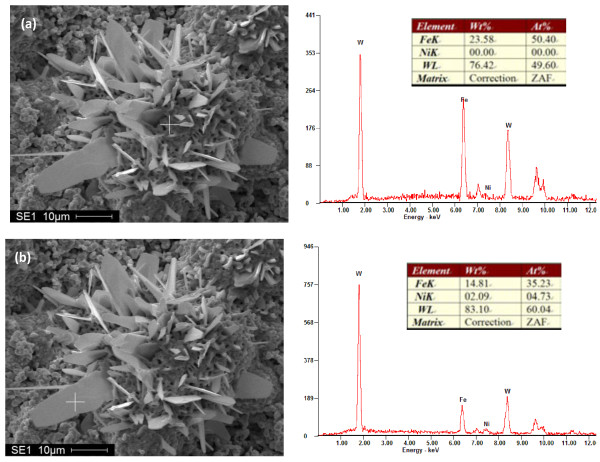
**EDS patterns of the Fe element segregation structures formed in the experiment.** (**a**) The aggregation structure formed by sintering; (**b**) the flake structure formed by Fe element segregation. ZAF, Z is atomic number correction factor, A is absorption correction factor, and F is fluorescence correction factor.

Figure4 shows the effect of Fe element on morphology of the flake-like structure . It implied that Fe element was benefit to the nucleation of tungsten flakes, and the aggregation formed by sintering possessed the highest content of Fe element, namely 23.58 wt.%. From bottom to top of the flake, the content of Fe element reduced gradually. Otherwise, the content of the Ni element only appeared a little.

**Figure 5:**
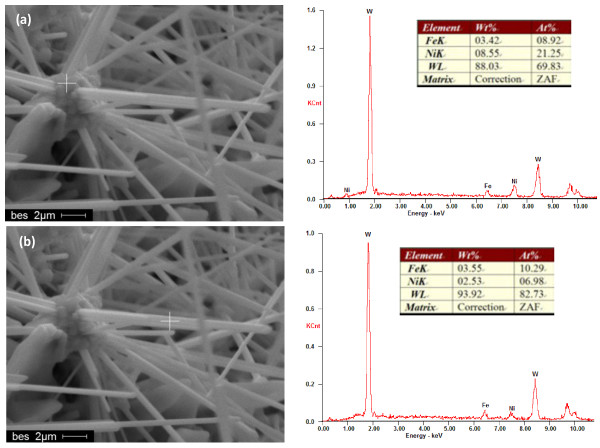
**EDS patterns of the Ni element segregation structures.** (**a**) The sintered aggregation structure; (**b**) the body of tungsten whisker-like structure. ZAF, Z is atomic number correction factor, A is absorption correction factor, and F is fluorescence correction factor.

During the experiment, besides the segregation of Fe element, the segregation of Ni element also existed. Figure5 shows a typical structure in which the whisker-like tungsten grew from the Ni element segregation configuration and the EDS patterns of different proportion. It showed that the content of Ni element reduced gradually from the bottom to the upper of the whisker-like structure. The aggregation structure resulted from melt containing the higher content of Ni element. The tip of the whisker had the lower content of Ni element, and at the same time, the content of Fe element changed a little. It implied that Ni element was benefit to the nucleation of tungsten whiskers.

**Figure 6:**
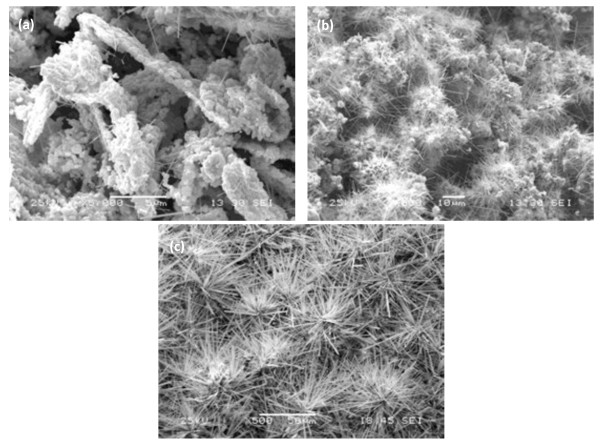
**Typical flower-like structures formed in growing process of whisker-like tungsten for different times.** (**a**) Nanowire-like structure by holding for 3 h; (**b**) initial large-scale tungsten whisker-like structure by holding for 4.5 h; (**c**) large-scale flower-like tungsten whisker structures by holding for 6 h.

**Figure 7:**
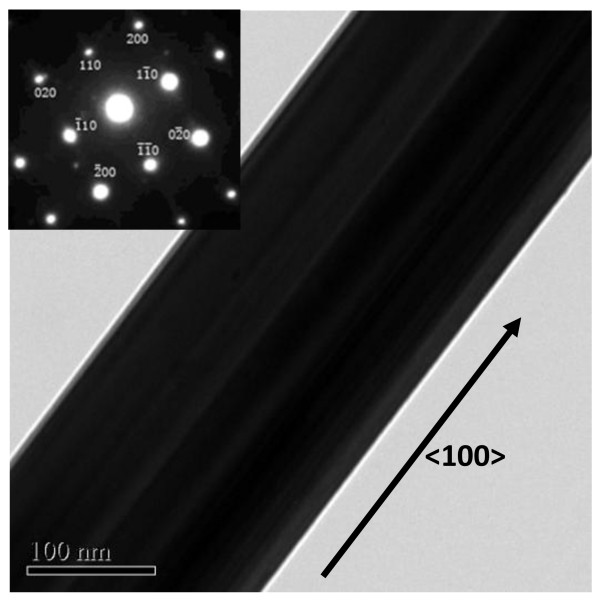
**The TEM image of tungsten whisker-like structure.** Inset: SAED pattern of tungsten whisker-like structure.

Figure6 shows a series of images of different structures during the growing process of the tungsten whisker-like structure for different times. Figure6a shows some imperceptible tungsten nanowires which grew from sintered powder by holding for 3 h; Figure6b shows an initial large-scale tungsten whisker-like structure by holding for 4.5 h; and Figure6c shows large-scale flower-like tungsten whisker structure caused by growing along different grains in the same pre-sintering aggregation by holding for 6 h. Figure7 is the TEM image and SAED of the tungsten whisker-like structure with the axial growth along <100 > direction.

### Growth mechanism

It is well known that the reaction between W and humid H_2_ is a complex process. Much research had been made about this process. Reaction with water vapor or humid H_2_ in the temperature range from 20°C to 500°C results in the formation of a WO_3_ layer. The thickness of the oxide layer increases with the increase of temperature. When the temperature increases to 500°C or 600°C, it can be severely oxidized to form WO_3_, which starts to evaporate with the formation of WO_2_(OH)_2_. At the temperature > 600°C, gaseous WO_2_(OH)_2_ is the most volatile compound formed in system W-O-H. It is responsible for all vapor-phase transport processes [24]. When the temperature reaches to 800°C, it can form large quantity of volatile. In the high water vapor content, W can react with water vapor directly, which can be expressed in the following:

(1) $$\text{W}\left(\text{s}\right)+{4\text{H}}_{2}\text{O}\left(\text{g}\right)\to {\text{WO}}_{2}{\left(\text{OH}\right)}_{2}\left(\text{g}\right)+{3\text{H}}_{2}\left(\text{g}\right)$$

The reaction of oxides of tungsten with water vapor can be expressed as follows:

(2) $${\text{WO}}_{3-x}\left(\text{s}\right)+\left(x+1\right){\text{H}}_{2}\text{O}\left(\text{g}\right)\to {\text{WO}}_{2}{\left(\text{OH}\right)}_{2}\left(\text{g}\right)+x{\text{H}}_{2}\left(\text{g}\right)$$

The formation and decomposition process of volatile compounds of WO_2_ (OH)_2_ can be expressed as in the equation below:

(3) $${\text{WO}}_{2}{\left(\text{OH}\right)}_{2}\left(\text{g}\right)+{3\text{H}}_{2}\left(\text{g}\right)\Leftrightarrow \text{W}\left(\text{s}\right)+{4\text{H}}_{2}\text{O}\left(\text{g}\right)$$

According to van’t Hoff isotherm, Gibbs free energy of Equation 3 can be calculated as follows:

(4) $${\text{Δ}}_{\text{r}}\text{G}=\Delta_{r}G^{\text{Θ}}+\text{R}TlnQ=-\text{R}TlnK_{\text{p}}+\text{R}TlnQ=-\frac{4}{3}\text{R}Tln\frac{P_{{\text{H}}_{2}\text{O}}^{\text{Θ}}}{P_{{\text{H}}_{2}}^{\text{Θ}}\cdot P_{{\text{WO}}_{2}{\left(\text{OH}\right)}_{2}}^{\text{Θ}}}+\frac{4}{3}RTln\frac{P_{{\text{H}}_{2}\text{O}}}{P_{{\text{H}}_{2}}\cdot P_{{\text{WO}}_{2}{\left(\text{OH}\right)}_{2}}}$$

where $\Delta_{r}G$ is the Gibbs free energy diversification of the reduction system; $\Delta_{r}G^{\Theta }$ is the Gibbs free energy at the standard state; R is constant, equal to 8.314 J·K^−1^·mol^−1^; *T* is reaction temperature; *Q* is pressure ratio of the materials at actual conditions; *K*_p_ is equilibrium constant; $P_{{\text{H}}_{2}\text{O}}^{}$$P_{{\text{H}}_{2}}^{}$, and $P_{{\text{WO}}_{2}{\left(\text{OH}\right)}_{2}}^{}$ are the partial pressures of H_2_O, H_2_ and WO_2_(OH)_2_, respectively.

The oxidation of W by water vapor and the reduction of WO_3_ by H_2_ are reversible processes. The direction of reaction can be changed by adjusting the partial pressure ratio of [H_2_O]/[H_2_] and further affects the growing process of tungsten whisker-like structure. For the presence of volatile compounds of WO_2_(OH)_2_ in the chemical reaction, the reaction atmosphere has a significant impact on the final product. The premise of ensuring $\Delta_{r}G\leq 0$, increasing partial pressure ratio of water vapor and hydrogen, is conductive to the generation of many volatile compounds of WO_2_(OH)_2_,which is the origin of fabrication of tungsten whiskers by vapor phase transmission and reduction of hydrogen.

**Figure 8:**
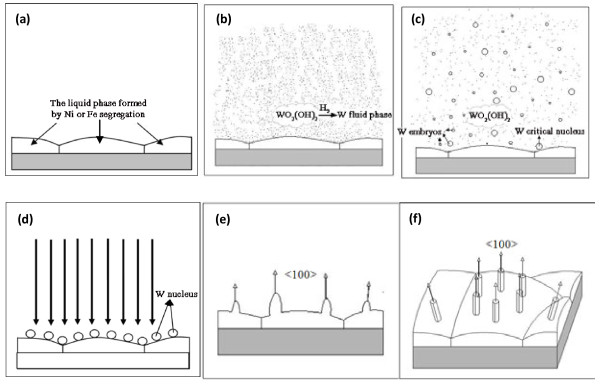
**The sketch map of the growth process of tungsten whisker-like structure.** (**a**)The liquid phase formed by Ni or Fe segregation; (**b**) tungsten fluid phase; (**c-d**) nuclear of tungsten formed by the agglomeration of tungsten atoms; (**e-f**) the growth of tungsten whisker-like structure.

According to above-reported results, the most distinct characteristic of the formed whisker-like structure is that the whisker-like tungsten has Ni-enriched structure at the bottom. We summarized the growth process of flower-like tungsten whiskers as follows: firstly, the W powder mixed with Ni and Fe was oxidized by water vapor, which would release lots of heat. The produced heat would bring the local temperature up to the melting point of Ni and Fe, this would lead to the formation of little liquid phase area, and the melting heat with the melting process would improve the temperature of the liquid system. The emergence of liquid phase caused the segregation of Ni or Fe element in some part of the system (Figure8a). Secondly, in the atmosphere of H_2_, gaseous WO_2_(OH)_2_ produced by the oxidation of tungsten and tungsten oxide by water vapor will be reduced and decomposed to form tungsten fluid phase with highly free energy (Figure8b). This fluid phase was a kind of metastable phase, it tended to form crystalline phase to reduce its Gibbs free energy. So, driven by the phase transition force, the tungsten fluid phase cohered to form tungsten embryos (Figure8c). The tungsten embryos whose radius increased up to the critical nucleus radius will deposit at the site with appropriate liquid concentration of Ni and Fe to form tungsten nuclei (Figure8c,d). The newly formed tungsten atoms in fluid phase will be adsorbed and deposited preferentially at the crystal plane of <100 > because of the difference of different crystal face in surface energy. Then, tungsten atoms diffuse along the side to the top, finally integrate with the top of the nanocrystal via the homoepitaxial growth, and thus lead to the axial growth along the <100 > direction (Figure8e). Under the guidance of crystal defects and high surface energy, the tungsten whisker-like structure was generated through pro-plane growth. During the growing of axial direction, the radial direction expanded at the same time because some tungsten atoms lingered around. The vapor phase transmission W atoms will be adsorbed preferentially on the sides and the tops of the W <100 > nanocrystallite that protruded from the W substrates (Figure8f). From Figures 1, 4, 5 and 6, it was very clear that mixing powder was pre-sintering and came into the aggregation, which included many small tungsten particles and little local liquid phase areas. Every small liquid phase area exposed to vapor phase may form tungsten whisker-like structures according to the above description. So, flower-like tungsten whiskers formed on the aggregation.

Due to the appearance of local high-temperature area, Ni and W can come into little solid solution (Ni_4_W). Otherwise, because of a considerable solid solution of W in Ni (about 16 at%) at 950°C and the very low solid solubility of Ni in W (about 0.05at%) [24], W atoms may dissolve into Ni particles to form solid Ni-W alloyed particles, and it is easy to reach saturation. W atoms diffuse through the solid Ni-W particle to form the nucleus of pure W nanocrystal. The subsequent W atoms integrate directly with the preformed W <100 > layer and result in the formation of a protrudent W <100 > whisker-like structure.

According to above description, the growth procedure of flower-like tungsten whiskers is probably based on the VLS mechanism at beginning of the formation of tungsten nuclei, that is, the VLS stage is from Figure8a-d. After that, vapor-solid mechanism is dominant (Figure8e,f).

## Conclusions

In summary, a novel route with low-cost to large-scale flower-like submicrometer-sized tungsten whiskers were fabricated by the vapor phase deposition method with the aid of metal catalysts. A large-scale submicrometer-sized tungsten whisker-like structure had the axial growth along the <100 > direction with large aspect ratio and possessed flower-like structure grown from the aggregation formed by sintering of W powder mixed with Ni powder. The growth procedure of flower-like tungsten whiskers is probably based on the VLS mechanism at beginning of the formation of tungsten nuclei. After that, vapor-solid mechanism is dominant.

## Competing interests

The authors declare that they have no competing interests.

## Authors’ contributions

YM carried out the experiments, participated in the sequence analysis and drafted the manuscript. JL carried out the partial experiments and participated in the mechanism analysis. WL participated in the design of the study and performed partial analysis. YS participated in the partial experiments. All authors read and approved the final manuscript.
